# The Role of Quality Improvement Projects in a Complex Abdominal Wall Service

**DOI:** 10.7759/cureus.48833

**Published:** 2023-11-15

**Authors:** Sofia Bitsios, Gaurav Kulkarni, Raunaq Chhabra

**Affiliations:** 1 Surgery, Mid and South Essex NHS Foundation Trust, Chelmsford, GBR; 2 General Surgery, Mid and South Essex NHS Foundation Trust, Chelmsford, GBR

**Keywords:** progressive pneumoperitoneum, quality improvement projects, complex abdominal wall reconstruction, hernia, qip

## Abstract

Background

Complex abdominal wall hernias have proven challenging to manage, and such patients often require abdominal wall reconstruction (AWR). However, in the context of a socialist healthcare service, which is required to provide equal and fair healthcare access to all, the heavy resource burden and non-life-threatening nature of complex abdominal wall hernias mean that this patient group may not be prioritised. In this paper, we outline the significant quality of life (QoL) burden on patients requiring AWR and the importance of quality improvement projects (QIPs) in establishing and streamlining their care as a robust, transferable model across centres.

Methodology

We undertook the creation of a regional AWR multidisciplinary team meeting and referral proforma, establishing a joint clinic between the Plastics and General Surgery teams and registering a standard operating procedure for the use of progressive pneumoperitoneum in a subset of AWR patients. We collected qualitative data using questionnaires sent out to clinicians and patients as well as used recognised outcome scales (pre- and post-operative European Hernia Society Quality of Life score, otherwise known as EuraHS-QoL score, and post-operative Carolinas Comfort Scale score) to assess responses to QIPs.

Results

Both clinicians and patients reported positive feelings towards the implemented changes, and scores following progressive pneumoperitoneum showed significant improvement.

Conclusions

Therefore, we propose that QIPs have a significant role in the establishment and streamlining of services for patients requiring AWR. Through the repeated use of QIPs, a robust, transferable model could be produced, which could then be shared with other regional specialist centres nationwide. As such, effective care could be offered equally to AWR patients for improved outcomes and reduced strain on healthcare resources.

## Introduction

Complex hernias have proven a challenge to socialist healthcare systems and abdominal wall surgeons alike. What constitutes a complex hernia has remained ill-defined, although a recent Delphi Consensus from the European Hernia Society has sought to elucidate this [1]. As such, it is also challenging to determine their prevalence. Despite this, the issue of complex abdominal wall hernias has long been recognised clinically and in research. Standard surgical approaches to complex hernias often lead to suboptimal outcomes in terms of aesthetics, recurrence, and quality of life (QoL) [2]. These outcomes are so well recognised that a phone application, the Carolinas Equation for Determining Associated Risk (CeDAR) application (by Carolinas Institute), has been developed on a database of thousands of previous patients to outline expected complication rates in AWR patients [3].

A growing body of evidence has shown that abdominal wall hernia patients benefit from abdominal wall reconstruction (AWR). This is the process by which surgeons attempt to recreate the abdominal wall, often via mesh reinforcement of the fascia at the midline. There are various methods by which AWR can be undertaken, with the ultimate goal of restoring structure and function [4]. However, AWR requires a highly specialised skillset and substantial resource allocation. As such, patients requiring AWR are often overlooked in socialist healthcare systems, given the non-life-threatening nature of their condition.

Despite their relatively benign status, complex hernias carry a heavy QoL burden [5], including repercussions on mental health, disruption of daily activities of living, and recurrent bowel obstruction [6,7]. Consequently, AWR patients often display a higher rate of comorbidities [8], thus further complicating their care while the root cause of their issue remains unaddressed. Caring for them, therefore, necessitates robust systems in specialist centres to break this cycle of poor health.

Specialist AWR centres in the United Kingdom are relatively new and scarce, often lacking clear referral pathways and surgical protocols. Clear pathways and protocols will allow surgeons to implement the growing evidence for AWR into their practice and offer timely and appropriate management [9,10]. Quality improvement projects (QIPs) are well adapted for this purpose, as they are often used to demonstrate the benefits of new approaches in practice. They do so by using the plan-do-study-act (PDSA) cycle to identify a clear goal and implement changes related to this goal. They then study the effect of the change and use their results to produce a meaningful difference in the service [11]. QIPs can be used for large-scale changes; however, they can also be used on a smaller scale to produce a meaningful difference in the patient experience. It is this latter use of QIPs that we aim to focus on in the current study. The evidence of how to surgically manage AWR patients has existed for some time [12,13]. However, their journey through the healthcare system remains a challenge. By introducing a clinical change via QIPs, and then taking into consideration the views and outcomes of the patients and clinicians who interact with these changes day-to-day, we are able to create a service that is ideally catered towards a complex patient subset, thus hopefully encouraging positive QoL changes.

With such considerations in mind, we formulated three QIPs and created a regional multidisciplinary team meeting (MDT) catered specifically to patients with complex abdominal wall issues. These QIPs ran concurrently as we sought feedback from the individuals involved. The first QIP was the creation of a formal referral proforma for use by other clinicians and hospitals to refer to the MDT and specialist service. Before this, there was no formal referral process. Feedback was then sought from the referring clinicians via feedback forms one year after implementation of the referral proforma. The second QIP involved the creation of a monthly joint clinic between the Plastics and General Surgery teams for the assessment of patients with complex abdominal wall issues. Feedback was collected from patients regarding their experience to justify the continuation of the joint clinic, as opposed to two separate clinics. The third QIP involved the creation of a standard operating procedure (SOP) for the use of Botox and progressive pneumoperitoneum in a specific subset of patients with massive incisional hernias, meeting the criteria for significant loss of domain. To our knowledge and based on a literature search, no SOP exists for this approach, and only level three evidence or lower has been generated for its use thus far. Its use in our hospital would require substantial resource allocation, with daily specialist input. As such, it was felt that implementing the SOP via a QIP into routine practice would allow more clinicians to become safely involved. This would allow pre-operative Botox and progressive pneumoperitoneum to be offered to more patients meeting the criteria.

Having outlined the needs of complex abdominal wall patients, the methods and uses of QIPs, and the QIPs implemented in our local hospital in Essex, United Kingdom, we hope to demonstrate the benefits of using QIPs in the care of complex abdominal wall patients requiring AWR.

## Materials and methods

All QIPs were run concurrently as prospective observational studies. The group of interest was identified, a change was implemented, and then their response to that change was assessed via qualitative methods. All QIPs occurred in a district general hospital within the National Health Service (NHS) in Essex, United Kingdom. The hospital serves a population of over 380,000, as per the previous Care Quality Commission report [14].

A monthly MDT was introduced in March 2022, specifically directed towards the discussion of complex abdominal wall patients. Any clinician from any hospital in the region could refer to this MDT. This included doctors and advanced nurse practitioners of any level and any speciality from primary and secondary care across Essex. Most referrals were from doctors and advanced nurse practitioners in General Surgery within the Mid & South Essex NHS Trust. However, no formal pathway existed to refer to this MDT, and the process was based on dictated letters that often did not convey the minimal information required for triaging or booking. Therefore, an official referral proforma was created (Appendix 1) in March 2022 as part of a QIP. This QIP was registered with the Audit and QIP Department at Broomfield Hospital (Mid & South Essex NHS Trust). The QIP underwent the local department’s ethical approval process. The referral proforma was distributed to all clinicians who had referred to the service in previous years, identified via their previous referrals. A year following the introduction of the proforma, a feedback form was distributed to gain qualitative data on clinicians’ experience of the new referral process (Appendix 2). A scale of one (strongly disagree) to five (strongly agree) was used to assess the level of agreement with a series of six statements with an area for comments at the end. These statements explored whether the proforma was easy to find and fill out, whether it saved time, and whether it was clear to whom it should be sent. The statements also explored whether clinicians felt that the referral proforma made it easier to refer to the complex abdominal wall MDT, improved the time for being seen, and improved patient and care experience, and whether they would preferentially use this referral system again.

Another QIP explored the patient experience of the service itself. A joint bimonthly clinic was introduced between the Plastics and General Surgery teams for the assessment of all complex hernia patients. No such service had existed before, and patients were assessed in each individual speciality’s clinic. The idea behind the introduction of the joint clinic was to allow a collaborative approach towards complex patient management and streamline the process for patients. A joint clinic meant fewer trips to the hospital and the ability to have specific questions answered by the appropriate speciality at the same time. This QIP was registered with the Audit & QIP Department at Broomfield Hospital and underwent local ethical approval as part of the QIP process. Formal ethical approval was not sought because anonymous patient responses were used only to assess the efficacy of the joint clinic. Feedback was sought anonymously using a form for which a quick-response (QR) code was provided in the clinic. No patient-identifying information was collected in this survey, and there was no use of clinical details or any change in a particular patient’s management based on feedback. Unfortunately, data could not be collected from before the joint clinic’s creation because no such joint clinic existed for comparison. Furthermore, there was no patient who had been seen in the individual clinics and the joint clinic. The feedback form consisted of a series of eight statements, with one additional area for comments (Appendix 3). As before, agreement with the statements was rated on a scale of one (strongly disagree) to five (strongly agree). The statements compared the joint clinic to previous clinic experiences, including whether patients felt their management was clearer and their questions were better answered by having both specialty consultants present and whether they overall preferred the joint clinic experience and would recommend it to others. To minimise bias, we only provided the feedback form to those patients who had prior documentation proving that they saw more than one specialty in different clinics in our trust for complex joint management of a specific health issue. We requested patients to answer the survey related to the joint clinic, drawing from their past experiences with other speciality clinics.

The third QIP established an SOP for progressive pneumoperitoneum so that it could be used safely and effectively by the wider General Surgical team, rather than being limited to the two individual specialists who were aware of its use. This was registered with the Audit & QIP Department at Broomfield and underwent the ethical approval process via the Medical Device Policy of Mid & South Essex NHS Trust (approval number 09032). Formal ethical approval was not sought because the process was not conducted as an experimental procedure but as an adjunct to repairing a complex hernia for which there were no available alternatives to increasing the intra-abdominal volume for loss of domain. Furthermore, the process was performed based on the work of other centres in the United Kingdom that offer this procedure and who have evidenced its benefits for patients meeting the criteria (i.e., it was not performed as a new experimental process) [12,15]. Based on the existing literature and after discussions with the few other centres in the United Kingdom that offer this procedure, we developed an SOP (Appendix 4, Appendix 5) to outline the process of Botox and pre-operative progressive pneumoperitoneum in the management of patients meeting the criteria of irreducible hernia and loss of domain >30%. To date, this SOP has been used on two patients who met the criteria, with good results clinically and in terms of standardised patient-reported outcomes measures using the Carolinas Comfort Scale (CCS) [16], Brehaut Decision Regret Scale [17], and European Hernia Society Quality of Life score (EuraHS-QoL) [18]. Both patients provided detailed informed consent before beginning the progressive pneumoperitoneum process. The EuraHS-QoL questionnaire was administered as a self-filled proforma in the pre-operative clinic, and then again in the clinic three months post-operatively to track patient-reported outcomes, as per the protocol for all AWR patients. The CCS was administered in the clinic at three months post-operatively. This is used purely post-operatively, as it is a questionnaire related to the sensation of mesh, and hence, is only appropriate for post-operative assessment. The SOP was followed by surgeons and nurses within the General Surgical team involved in the care of these patients, under the supervision of the specialists.

## Results

**Figure 1:**
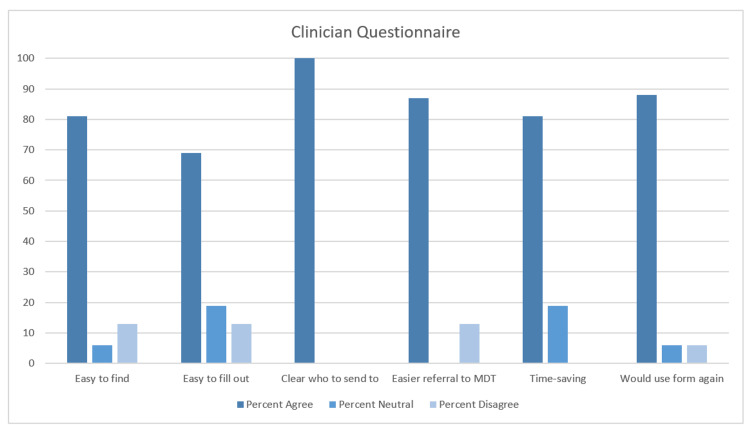
Percentage response (agree, neutral, disagree) to each question in clinician feedback.

Overall, feedback obtained from both clinicians and patients showed a positive response towards service changes. Of the 20 surgeons to whom the feedback form was sent, 16 (80%) responded. The majority showed a positive response (‘Agree’ or ‘Strongly Agree’) to the following statements: the referral proforma was easy to find (13 clinicians), the referral proforma was easy to fill out (11 clinicians), it was clear where to send the referral (16 clinicians), I feel this method of referral made it easy for me to refer to the complex abdominal wall hernia MDT (14 clinicians), having a clear referral process saved time (13 clinicians), and I would use this referral system again (14 clinicians). Table 1 and Figure 1 below set out the responses to each statement with percentages of responses in agreement (those responding agree and strongly agree), responses with neutral stances (those responding neutral), and responses in disagreement (those responding disagree or strongly disagree).

**Table 1:** Results from clinician feedback. MDT: multidisciplinary team

Statement	Strongly agree	Agree	Neutral	Disagree	Strongly disagree	Percentage agree	Percentage neutral	Percentage disagree
Referral proforma was easy to find	3	10	1	2	0	81%	6%	13%
Referral proforma was easy to fill out	6	5	3	2	0	69%	19%	13%
It was clear where to send the referral	8	8	0	0	0	100%	0%	0%
This method made it easier to refer to the MDT	8	6	0	2	0	87%	0%	13%
Having a clear referral process saved time	8	5	3	0	0	81%	19%	0%
I would use this referral system again	8	6	1	1	0	88%	6%	6%

**Figure 2:**
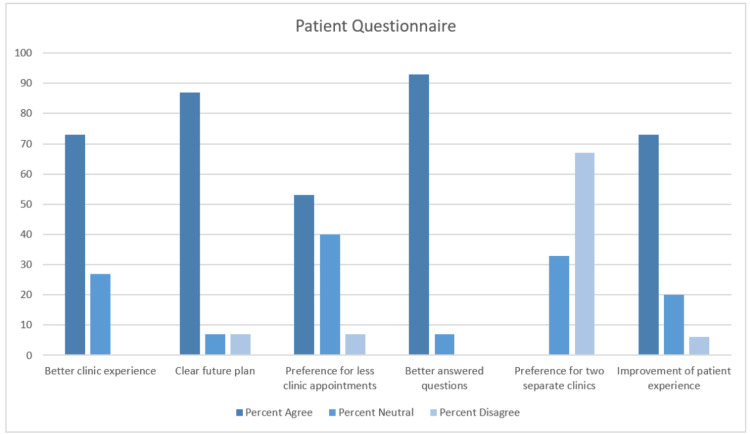
Percentage response (agree, neutral, disagree) to each question in patient feedback.

Of the 15 patients seen in the joint clinic to whom the feedback form was provided, all 15 (100%) responded. The forms were provided only to those patients seen in the joint AWR clinic who had required multiple appointments to see different clinicians for any specific health issue in the past. The majority showed a positive response (‘Agree’ or ‘Strongly Agree’) to the following statements: I found this clinic experience better than the previous one (11 patients); following my consultation, I feel there is a clear plan going forward (13 patients); I preferred having fewer clinic appointments to attend (eight patients); my questions were better answered by having consultants from both specialities present (14 patients); I would have preferred two separate clinics with each specialist instead of a joint clinic (zero patients); and, overall, I feel that the joint clinic has improved my patient experience (11 patients). Table 2 and Figure 2 below set out the responses to each statement, with percentages of responses in agreement (those responding agree and strongly agree), responses with neutral stances (those responding neutral), and responses in disagreement (those responding disagree or strongly disagree).

**Table 2:** Results from patient feedback.

Statement	Strongly agree	Agree	Neutral	Disagree	Strongly disagree	Percentage agree	Percentage neutral	Percentage disagree
I found this clinic experience better than the previous	4	7	4	0	0	73%	27%	0%
Following my consultation, I feel there is a clear plan going forward	6	7	1	0	1	87%	7%	7%
I preferred having fewer clinic appointments to attend	2	6	6	1	0	53%	40%	7%
My questions were better answered by having consultants from both specialities present	5	9	1	0	0	93%	7%	0%
I would have preferred two separate clinics with each specialist, instead of a joint clinic	0	0	5	2	8	0%	33%	67%
Overall, I feel that the joint clinic has improved my patient experience	4	7	3	0	1	73%	20%	7%

Two patients had undergone the Botox and progressive pneumoperitoneum protocol (Appendix 5) since its creation six months prior. Both patients met the initial criteria: loss of domain >30% and an irreducible hernia on clinical examination. Both patients recovered well post-operatively (Table 3). Pre-operative EuraHS-QoL scores were 57 and 60. Post-operatively, these scores improved to 22 and 25, respectively, indicating a marked improvement for both patients following the implementation of the SOP before the complex AWR. Post-operative CCS scores were 17/115 and 23/115, respectively. The Brehaut Decision Regret Scale administered anonymously to both patients revealed a score of 6 each, with 5 being the best and 25 being the worst score possible. These results indicate a good adjustment to the surgical process and the mesh used. Both patients continue to be followed up.

**Table 3:** EuraHS-QoL scores, CCS scores, and Brehaut Decision Regret Scale scores for both patients who underwent the pre-operative progressive pneumoperitoneum SOP. CCS: Carolinas Comfort Scale; EuraHS-QoL: European Hernia Society Quality of Life; SOP: standard operating procedure

Patient	EuraHS-QoL		CCS	Brehaut Decision Regret Scale
	Pre-operative	Post-operative		
Patient 1	57	22	17	6
Patient 2	60	25	23	6

## Discussion

The above outcomes demonstrate how QIPs can be used effectively in the care of complex hernia patients to introduce practice-based changes and measure their outcomes in a standardised manner. By recording positive outcomes, the use of novel evidence-based approaches can be justified in practice.

The introduction of a referral proforma and joint clinic were both met with overall positive feedback from the individuals interacting with those services. As such, the QIP outcomes could then be used to justify the change in practice and allow it to continue. Had feedback been negative, the changes could have been withdrawn and adjusted accordingly. Evidence-based approaches do not always translate well into real clinical practice. Therefore, by introducing them in this manner, the service is able to cater to a specific patient subset. This is of particular importance in complex patient subsets, as there are numerous confounding variables in their care which may occasionally be missed or underestimated in the literature.

The use of a QIP to introduce the progressive pneumoperitoneum SOP further highlights how QIPs can be used to justify certain approaches within a socialist healthcare model. Running the progressive pneumoperitoneum SOP requires a high allocation of resources. This resource allocation becomes particularly high if the protocol can only be run by specialists. The introduction of a standardised protocol provides a guide to which other clinicians and services can refer. It reduces the burden on resources by allowing members of the wider team to become involved by following the protocol. This can be performed under the supervision of the specialist, if necessary. The standardised protocol can be disseminated to other centres, allowing pre-operative progressive pneumoperitoneum to occur within local hospitals, with transfer to a specialist centre for operative and post-operative management. By allowing the pre-operative care to occur locally, the patient burden on the specialist centre is reduced, and patients avoid having to make frequent longer journeys. Furthermore, by using a QIP to demonstrate positive patient QoL outcomes following the implementation of the SOP, there is evidence to justify the high allocation of resources in the context of a non-life-threatening but nonetheless debilitating disease.

The successful use of QIPs for complex patient subsets has been demonstrated in several previous studies. It is already well known that patient feedback, collected by various staff members in formal (voluntary groups, forms) and informal ways, can be used to improve healthcare services [19,20]. In a review of the literature, all studies using QIPs were shown to have implemented at least one improvement [21], indicating their high rate of success. Qualitative feedback is so valued that socialist healthcare services such as the NHS have produced a framework by which to guide trusts to use the patient experience to improve services [22]. QIPs are also useful in improving working conditions for clinicians, as evidenced by the use of feedback to make referral processes and the required information easier to find [10], thereby improving both time effectiveness and the quality of referrals.

The use of QIPs in complex abdominal wall surgery has been less well studied, yet there is still evidence to prove its use. They have been used to guide intra-operative methodology in complex AWR [23], as well as in formulating clear clinical pathways to allow the introduction of the York Abdominal Wall Unit, which caters specifically to this subset of patients [9]. QIPs were also used over a three-year period to improve intra-operative techniques and post-operative care, resulting in reduced material cost, improved post-operative outcomes, and no mesh-related complications [24]. Thus, the evidence shows that the use of QIPs in complex abdominal wall patients can produce real-world changes that improve all aspects of their care.

However, most QIPs do not follow the accepted methodology, which can be a limitation and can prevent them from being used to their true potential. It is proposed that QIPs follow the PDSA cycle method to gain the most effective outcome [25]. However, in a systemic review of QIPs, which assessed methodology and reported outcomes, it was found that there was low adherence to the use of specific aims and PDSA cycles [26]. This reduced their effectiveness, legitimacy, and reproducibility. Therefore, there is a need to improve and standardise the QIP methodology to use it for service improvement and research.

The small sample size and limited geographical location of the QIPs in this paper could be a limitation; however, as demonstrated in previous reviews, even small sample sizes can show benefits. QIPs are most effective when large, infrequent projects are used in conjunction with smaller, more frequent projects [27]. The smaller projects can produce local benefits, which can then be used as evidence to justify larger-scale projects. This is particularly important in small, understudied patient populations, such as those requiring AWR, as larger QIPs can be difficult to justify as a part of equitable healthcare in the absence of clearly demonstrated benefits. Furthermore, QIPs performed on a larger scale have demonstrated that providers may become fixated on a certain measurement and, in doing so, demonstrate ‘crowding out’ behaviour. This is where the intended improvement in one area is implemented at the expense of care in another, which may not always align with patient preference [28]. Smaller-scale QIPs allow more one-to-one feedback between those undertaking the QIP and those participating in it, thus allowing for better real-time adaptations.

The QIPs outlined in this paper represent the start of a wider project. With evidence now supporting their use in complex abdominal wall patients, we aim to introduce QIPs at all levels of their care, to allow for integration of evidence-based approaches. We hope to achieve a model of care for AWR patients that is safe, effective, and transferable, thereby reducing recurrence rate and post-operative complications. In doing so, we might take forward the work in pathways and prehabilitation that have already been created through the York Abdominal Unit [9] and might extend such pathways nationwide. Creating a transferable model could allow for the establishment of other specialist centres in regions across the country, thereby improving awareness of the condition, and allowing equal access to specialist care.

## Conclusions

Complex abdominal wall patients have proven a challenge to manage. This is particularly the case in socialist healthcare models, in which resources must be allocated equally and fairly. In this study, we have evidenced the use of QIPs in the care of complex abdominal wall patients. We have shown that QIPs can be used to introduce changes to complex patient care, and then justify their ongoing use. We have also evidenced how QIPs can be used to justify high resource allocation in the context of a non-life-threatening disease. We recognise the limitation of not following appropriate methodology and therefore must be strict in our approach to produce optimal outcomes. We also recognise the limitation of small sample sizes; however, we believe that these could form the basis of larger-scale projects. In doing so, we might propose a robust, transferable model for the care of AWR patients that might improve their care and outcomes at every level.

## Figures and Tables

**Figure 3:**
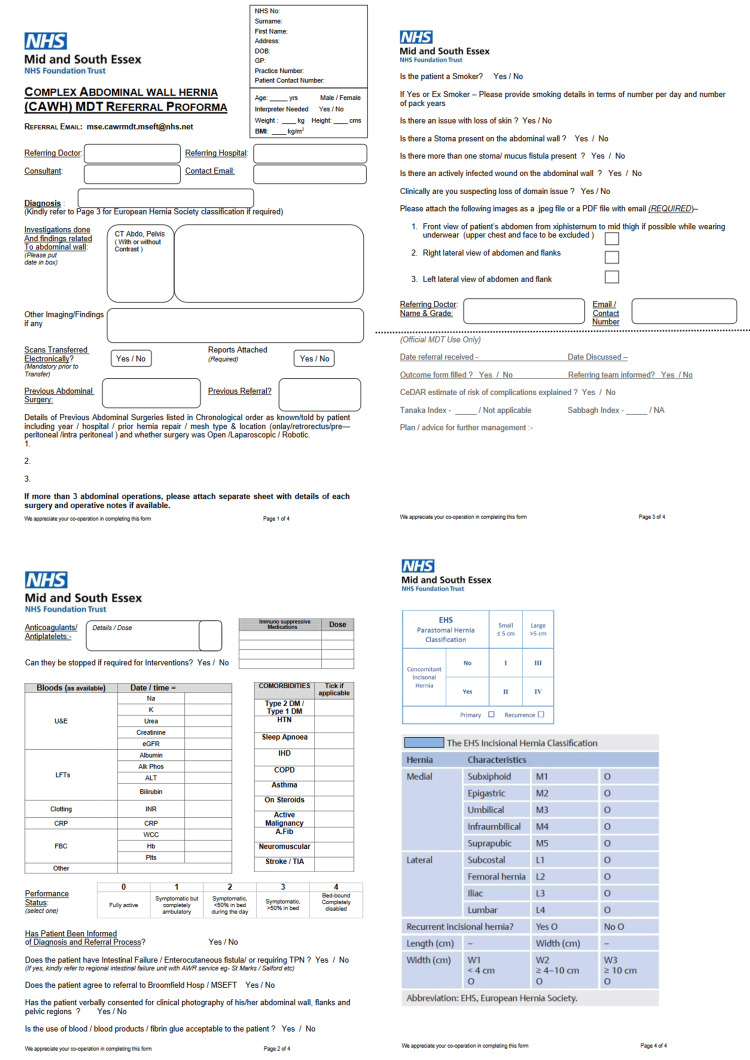
Complex abdominal wall hernia multidisciplinary team referral form.

**Figure 4:**
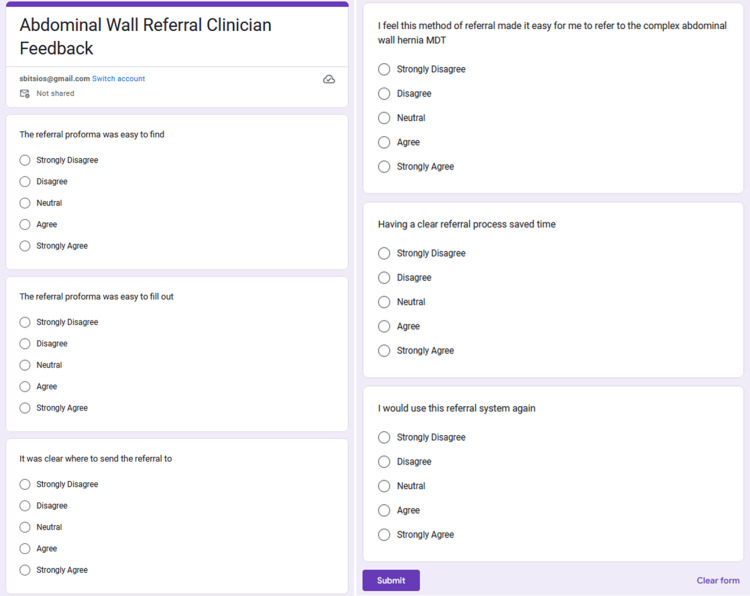
Clinician feedback form.

**Figure 5:**
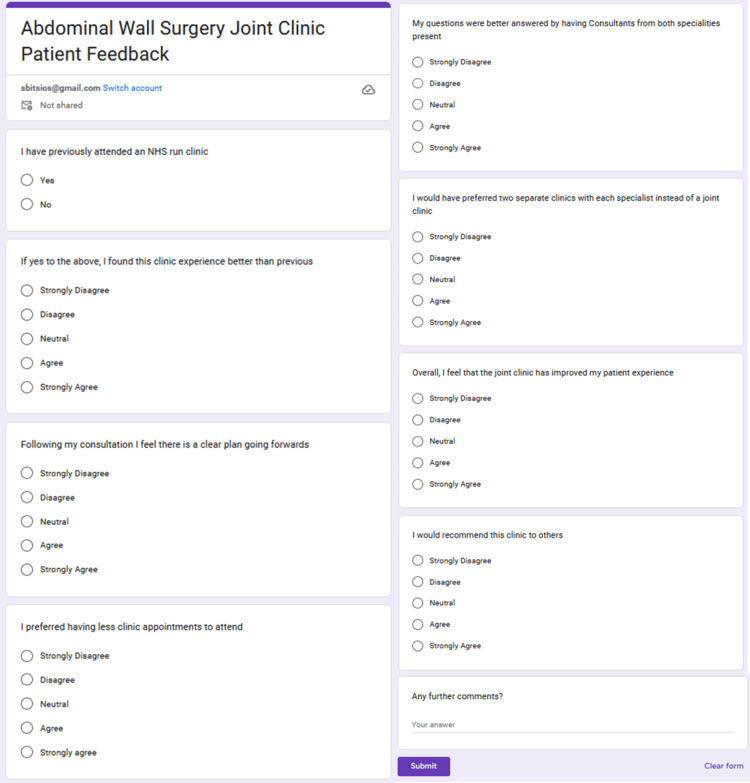
Patient feedback form.

**Figure 6:**
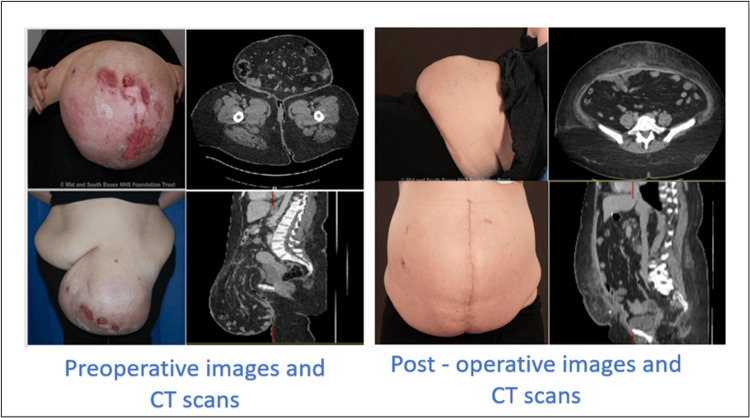
Pre and post-operative pictures and CT images for comparison of one patient who required progressive pneumoperitoneum (PPP) using the standard operating procedure created for the PPP protocol.

**Figure 7:**
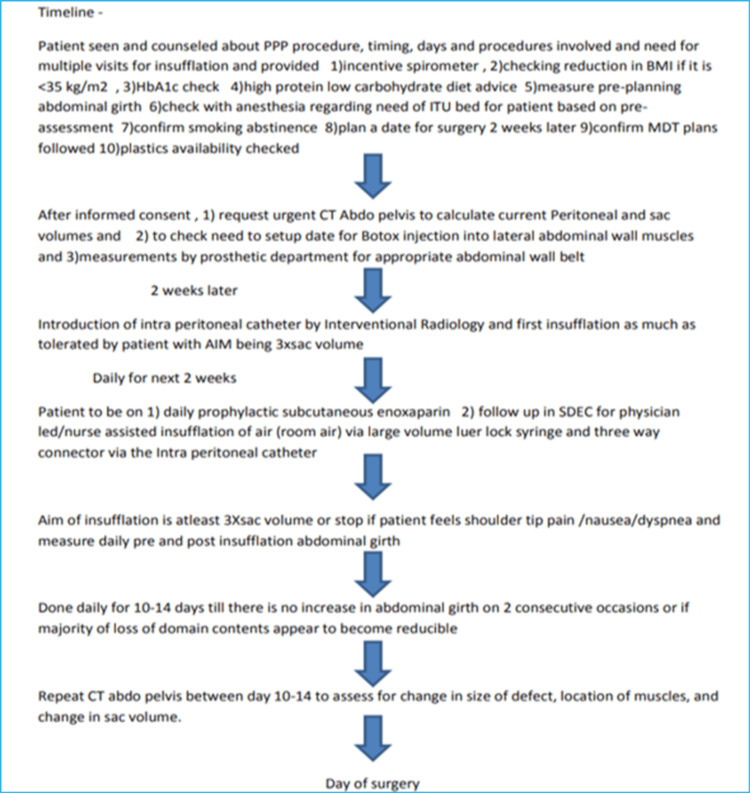
Preoperative progressive pneumoperitoneum (PPP) protocol flowchart.

## Appendices

Appendix 1

Appendix 2

Appendix 3

Appendix 4

Appendix 5
